# Shining a light on marginal food insecurity in an understudied population

**DOI:** 10.1017/S1368980022001094

**Published:** 2022-08

**Authors:** Angela D Liese

**Affiliations:** Department of Epidemiology and Biostatistics, Arnold School of Public Health, University of South Carolina, Columbia, SC 29208, USA

In this issue, Rabbitt *et al*. describe findings from a June 2020 Behavioral Health Epidemiological Consultation (BH-EPICON) survey conducted among active-duty Soldiers at an Army installation in the USA^(1)^. The survey retrospectively assessed household food insecurity experienced in 2019, prior to the onset of the COVID-19 pandemic, and during the first 6 months of the pandemic in 2020. The authors report a significant increase in the prevalence of marginal food insecurity, from 19 % in 2019 to 32·7 % in 2020. Further analyses of those reporting marginal food insecurity in 2020 revealed that about half of these households were food insecure at both time points, whereas the other half transitioned into marginal food insecurity with the onset of the pandemic.

For over 30 years, the US Department of Agriculture’s classification system for food security has remained unchanged. Although households affirming one or two of the eighteen items of the widely used Household Food Security Survey Module (HFSSM) questionnaire are conventionally considered to have ‘marginal food security’, this group is generally combined with those with ‘high food security’ (no HFSSM affirmations) into the group of ‘food secure’ households, whereas respondents affirming three or more HFSSM items are considered ‘food insecure’^(2)^. Given that the BH-EPICON survey administered a two-item food security screener instead of the entire HFSSM, Rabbitt *et al*.^(1)^ were essentially compelled to focus on marginal food insecurity, and a single affirmation of one of the two questions was sufficient for classification as marginally food insecure. This is fortuitous because growing evidence suggests that the consequences, including the adverse health outcomes of marginal food insecurity, parallel those of more severe levels of food insecurity^(3–6)^. In this context, the authors’ use of language is refreshing as they consistently use the phrase ‘marginal food *insecurity’ rather than* ‘marginal food *security*’ as labelled by the HFSSM scoring in the USA. Their much more intuitive label is reminiscent of the language (albeit not the specific methodology) used by the Canadian Community Health Survey^(7)^.

To date, very little research has been conducted on food insecurity in the US military. Previous work by Beymer *et al*.^(8)^ based on a 2019 survey at another military installation reported a 33 % prevalence of marginal food insecurity compared with the 19 % reported by Rabbitt *et al*. for that same year^(1)^. Using the conventional food security classification scheme, the 2020 Military Families Lifestyle Survey indicates that 14 % of active-duty military families were classified as having low food security or very low food security^(9)^. This number would have been substantially higher if the report had considered marginal food insecurity as well.

Rabbitt *et al*.’s analyses of the prevalence of marginal food insecurity before and during the pandemic suggest similar increases in marginal food insecurity in the Army as in a civilian comparison group from a national survey^(1)^. However, not all publications to date have shown pandemic-related food insecurity increases. For instance, the US Department of Agriculture’s 2020 food security report shows an increase between 2019 and 2020 in the prevalence of food insecurity in US households with children but not in the overall US population^(10)^.

Rabbitt *et al*.’s paper additionally evaluates a range of predictors for the transition into marginal food insecurity^(1)^. Characteristics that were found to be predictive included being single (*v*. married), self-identified race and ethnicity reported as Asian/Pacific Islander or Black (*v*. non-Hispanic White), military rank of enlisted Soldiers ranging from Private to Chief Warrant Officer (*v*. officer rank), higher post-traumatic stress disorder scores, a larger number of dependents, financial insecurity of the Soldier and their spouse and concerns about family members’ job security. Interestingly, characteristics such as age, sex, educational attainment, levels of social support, depression and anxiety were not independently predictive. In 2020, 481 000 of the roughly 1·3 million US active-duty service members were with the Army, and of these, about 81 % were enlisted Soldiers, which is the population most represented in the paper by Rabbitt *et al*.^(11–13)^ More than half of Army Soldiers were married, 85 % were male and more than 40 % had children. In this context, the findings that any of the ranks of enlisted Soldiers were independently associated with an increased risk of transitioning from food security into marginal food insecurity and that single marital status was the only characteristic associated with decreased risk underscore the importance of the spouses’ or family members’ roles in income generation. This is further supported by the findings that indications of financial insecurity and worry about the job security of a family member were similarly associated with increased risk of transition into marginal food insecurity^(1)^.

Food insecurity is thought to affect health through nutritional, behavioural and mental health pathways, of which the latter two are most likely of particular relevance to active-duty service members^(14)^. Food insecurity has been consistently associated with substantially increased levels of stress and depression, as well as with anxiety in North American populations^(15)^. Considering the inherent stress and sacrifices associated with active-duty military service, the experience of household food insecurity and its consequences places a substantial number of the enlisted Soldiers at a serious disadvantage.

Moreover, food insecurity’s impact extends well beyond the individual service member’s and household’s health and well-being. Experiences of food insecurity are likely to undermine a Soldier’s resilience and diminish their ability to recover fully from physical or mental injury sustained while on active duty. Food insecurity may also severely impact a Soldier’s willingness and ability to continue serving their country. Previous work by this author group has in fact provided a first line of evidence to this point. Beymer *et al*.^(8)^ demonstrated that marginal food insecurity was associated with poor mental health outcomes including suicidal ideation and related to intentions to leave the Army^(8)^. Last but not least, children of enlisted Soldiers in food insecure households will likely experience many of the same disadvantages associated with childhood food insecurity in civilian populations^(16)^.

In summary, Rabbitt *et al*.’s results of a third of surveyed active-duty households at one Army installation being marginally food insecure highlight an issue that merits study across all branches of the US armed forces. While the authors caution that their findings should be interpreted as a likely upper bound of the extent of the food insecurity problem, any level of food insecurity in the US military should be a cause for grave concern for all of us, civilians, military families and service members alike, because food insecurity undermines service members’ readiness. Policymakers should act rapidly to alleviate the few known barriers to food assistance eligibility of active-duty Soldiers while more systemic solutions are being developed.
